# A lightweight remote sensing small-object detection approach with scale-based dynamic loss and efficient multi-scale attention

**DOI:** 10.1038/s41598-026-55239-9

**Published:** 2026-06-04

**Authors:** Anying Xu, Xuanyu Wu, Wuzhong Yang

**Affiliations:** 1 School of Mechanical Engineering, Guizhou University, Guiyang, 550025 China; 2 State Key Laboratory of Fluid Power and Mechatronic Systems, Zhejiang University, Hangzhou, 310027 China

**Keywords:** Engineering, Mathematics and computing

## Abstract

Accurate small-object detection is crucial for automated processes in remote sensing imagery, such as environmental monitoring and aerial surveillance. Yet it remains challenging due to limited feature representation, complex backgrounds, and localization errors. To address these challenges, this work proposes a lightweight detection approach that integrates a scale-based dynamic loss and efficient multi-scale attention. A lightweight backbone, dubbed A2C2fLite, is designed based on the A2C2f module from the most recent YOLO variant to reduce redundant parameters and computational overhead. An EMA module is embedded before the final detection heads to enhance multi-scale feature representation and improve target-background discrimination. The SD loss is used to effectively address scale variation and occlusion, improving localization accuracy beyond existing Intersection-over-Union-based losses. Evaluated on a representative remote sensing dataset, the proposed approach achieves 93.6% precision, 86.6% recall, and 92.6% $\hbox {mAP}_{50}$, with only 2.0M parameters and 5.7 GFLOPs. Compared to the baseline YOLOv13, it achieves a superior accuracy-efficiency trade-off by reducing parameter counts by 20% and computational overhead by 8.1%, while improving $\hbox {mAP}_{50}$ by 1.5%. Visualization results demonstrate that it suppresses missed alarms and refines bounding-box localization. By minimizing memory footprint and computational bottlenecks, this lightweight design provides clear practical advantages for real-time small-object detection on resource-constrained edge devices, such as onboard Unmanned Aerial Vehicle systems and embedded aerial sensors. These findings highlight that the proposed approach offers an efficient, robust, and highly deployable solution for complex remote sensing applications.

## Introduction

Small object detection in remote sensing imagery has emerged as a fundamental yet challenging task in computer vision, with critical applications in urban planning, disaster monitoring, maritime surveillance, and environmental protection^1,2^. Unlike targets in natural scenes, objects in remote sensing images (e.g., vehicles, ships, and storage tanks) are often captured from a bird’s-eye view, leading to two distinct challenges: extreme scale variation and arbitrary orientation^3^. First, targets typically occupy only a few dozen or even fewer pixels within a high-resolution wide-swath image. This high scale sensitivity means that even a minor localization shift can result in a dramatic drop in intersection over union (IoU), causing traditional detectors to fail in precise localization. Second, remote sensing targets are distributed with arbitrary orientations. Conventional horizontal bounding box detection often introduces redundant background noise and encounters severe overlaps in densely packed regions, such as parking lots or crowded harbors.

In recent years, deep learning–based object detection models^4,5^ have demonstrated remarkable performance improvements in a wide range of visual recognition tasks. Among them, convolutional neural networks (CNNs)^6–8^ have been widely adopted for feature extraction and multi-scale object detection. Object detection algorithms are broadly categorized into one and two-stage methods. The latter, e.g., R-CNN^9^ and faster R-CNN^10^, provide high accuracy but suffer from high computational cost and slow inference^11^. Sagar et al.^12^ propose a novel remote sensing scene understanding system called multiscale-attention R-CNN. It achieves a mean average precision (mAP) of 74.37 % on the DIOR dataset when the gamma value is set to 0.2 and 81.97 % on the DOTA dataset when the gamma value is set to 0.1 with the same learning rate, outperforming state-of-the-art models on both datasets. The former, such as Single Shot MultiBox Detector (SSD)^13^ and You Only Look Once (YOLO)^14^, can achieve a better trade-off between speed and accuracy than the latter. The YOLO series^15–22^, in particular, has gained widespread adoption in real-time detection tasks owing to its high efficiency and strong feature representation ability. Cheng et al.^23^ introduce YOLO-World that enhances YOLO with open-vocabulary detection capabilities through vision-language modeling and pre-training on large-scale datasets. It achieves 35.4 AP with 52.0 FPS on V100 and outperforms many state-of-the-art methods in terms of both accuracy and speed on the challenging LVIS dataset. Recently, YOLOv13^24^ has emerged as a powerful baseline due to its optimized architecture and improved detection accuracy, making it highly suitable for real-time small-object detection.

Building on the success of these high-performance and efficient detectors, researchers have examined such key design aspects as lightweight backbones, attention enhancements, and refined localization losses. They must be jointly optimized to push YOLO-class models toward accurate small-object recognition without sacrificing real-time speed. Lightweight network design has been extensively explored to meet the efficiency requirements of object detection in resource-constrained environments^25^. Such models as MobileNet^26^, ShuffleNet^27^, and GhostNet^28^ reduce computational cost and parameter size through depthwise separable convolutions, channel shuffling, or linear transformations. However, they often compromise feature representation capacity, which is particularly detrimental for small-object detection where fine-grained details are critical. For example, when detecting small vehicles or ships, lightweight models may fail to capture edge and contour information, leading to missed detections^29^. To mitigate this loss in representational power, attention mechanisms have been widely adopted as a complementary strategy to improve feature representation in object detection^30^. Such modules as Squeeze-and-Excitation Attention (SE)^31^, convolutional block attention module (CBAM)^32^, and coordinate attention (CA)^33^ enhance feature selectivity by focusing on informative channels or spatial regions. Despite their effectiveness, many of these designs incur high computational cost and may not generalize well in cluttered scenes with multiple small targets. In small-object detection tasks, targets that occupy only a few pixels–such as distant pedestrians, tiny fasteners, or remote traffic signs–are easily confused with the surrounding texture when the attention module is not scale-aware, leading to frequent false positives. While better features improve discrimination, accurate localization remains equally critical. Hence, bounding box regression has received growing attention. Conventional IoU-based losses, including generalized-IoU (GIoU)^34^, distance-IoU (DIoU)^35^, and complete-IoU (CIoU)^36^, have been proposed to improve optimization, yet they often fail to handle scale variation and partial occlusion effectively. These limitations are particularly evident in small-object detection, where even slight deviations in predicted boxes can cause severe performance degradation. For example, when two small ships partially overlap in aerial imagery, IoU-based losses tend to fail to provide sufficient guidance to achieve precise localization.

This work proposes a lightweight remote sensing small-object detection approach based on scale-based dynamic loss (SD loss)^37^ and efficient multi-scale attention (EMA)^38^. The EMA module adopts a multi-branch structure to capture long-range spatial dependencies and cross-scale context. By enhancing the model’s spatial perception, EMA allows the network to learn more robust geometric features, which helps in identifying target structures regardless of their arbitrary orientation. Simultaneously, the SD Loss addresses scale sensitivity by dynamically adjusting the optimization weights based on the target’s pixel area. By assigning higher priority to localization accuracy for smaller targets, the SD Loss ensures stable gradient propagation. The novel contributions this work aims to make are:

1. A lightweight A2C2fLite module is designed to reduce model complexity while maintaining effective feature representation for small-object detection.
2. An EMA module is incorporated into the neck to enhance feature refinement and improve target-background discrimination.
3. A SD loss is introduced by adapting an existing IoU-based formulation to improve localization robustness under varying object scales and occlusion conditions.
4. Based on the above components, we construct a lightweight small-object detection framework named LSE-YOLO.
5. The experimental comparison results on the NWPU VHR-10 dataset demonstrating that LSE-YOLO outperforms other object detection models and provides a reference for the real-time detection of small objects.

## Related work

### Lightweight models for small-object detection

Designing compact detectors that retain strong small-object representation is a core challenge for resource-constrained deployments^39^. Wang et al.^40^ propose LSOD-YOLO, which inserts a lightweight cross-layer output reconstruction (LCOR) module into YOLOv8 to strengthen shallow–deep feature fusion. Despite improved small-object recall, LCOR relies on three fixed-scale outputs, leaving finer layers under-exploited. Mahaur et al.^41^ embed a versatile pruning scheme into IS-YOLOv5 for in-vehicle inference. Their aggressive channel slimming, however, inevitably erases weak edge activations that are essential for tiny-instance localization. Nikouei et al.^42^ present L-CNN, replacing standard convolutions with depthwise-separable ones and cutting layer-wise filters for edge-level human detection. Yet its domain-specific pruning and lack of scale-aware or cross-channel recalibration cause visible performance drops once generalized to diverse small objects or cluttered scenes. Beyond architectural redesign, model-compression techniques, e.g., pruning, quantization and knowledge distillation, are widely adopted to reduce inference cost. Lyu et al.^43^ present a survey of object detection model compression techniques. They empirically show that, although these methods effectively shrink large object detectors, they often incur non-negligible accuracy drops on small-object instances, underscoring the need for more adaptive compression schemes. Many of them trade off fine-grained spatial detail needed for small targets: aggressive parameter reduction or overly shallow feature hierarchies can degrade localization and classification of small objects. Consequently, lightweight designs tailored for small-object scenarios must balance parameter efficiency with the mechanisms that preserve or recover discriminative features. In addition, although recent lightweight backbones have achieved considerable reductions in computational complexity and parameter size, many of them are primarily optimized for general object detection or classification tasks. As a result, they may insufficiently preserve fine-grained spatial information and weak semantic cues that are critical for small-object detection in UAV scenarios. Excessive simplification of feature extraction pipelines can also weaken shallow feature representation, thereby limiting detection performance in dense or cluttered environments.

### Attention mechanisms for small-object detection

Attention mechanisms have become crucial in small-object detection for enhancing feature discrimination and suppressing background noise. By focusing on relevant regions and channels, attention modules help models detect small objects in complex background. Among the various attention schemes, channel and spatial attention ones enhance feature selectivity, while coordinate and multi-scale attention ones are specifically designed to encode positional and scale information, which are particularly beneficial for detecting small targets. Li et al.^44^ propose a multi-head channel and spatial trans-attention module that performs remote pixel interaction from both channel and spatial dimensions, effectively capturing attention features. Their method aims to address the challenge of long-range dependencies while maintaining local spatial coherence. Gong et al.^45^ introduce a feature-aware detection framework built upon YOLOv8 by integrating multi-scale edge enhancement with light-guided attention mechanisms. It consists of three key modules: (1) multi-scale edge enhancement, which refines edge features in low-contrast environments; (2) light-guided attention, which adaptively modulates features across regions; and (3) partial convolution, which optimizes computational efficiency. In another approach, Veraar^46^ explores the potential of transformer-style attention and combine it with spatial-temporal fusion mechanics, using a Video Swin backbone. This approach aims to resolve shape mismatches between the backbone output and model input while preserving temporal information. While such transformer-based attention methods show promise, they often introduce significant computational overhead, posing a huge challenge for real-time and resource-constrained applications. In addition, many existing attention mechanisms focus predominantly on improving global contextual modeling, but often overlook the computational burden introduced by repeated feature recalibration and multi-branch aggregation. This issue becomes more pronounced in UAV scenarios where real-time inference and limited onboard resources are critical requirements. Some lightweight attention variants reduce complexity by simplifying channel interactions, yet such simplifications may weaken the model’s sensitivity to tiny or partially occluded objects. To address this limitation, research has shifted towards more efficient multi-scale attention methods that selectively enhance small-object signals without significantly increasing latency or computational cost. The EMA module^38^, which is adopted in this work, aims to strike a balance between attention refinement and computational efficiency, making it well-suited for lightweight detectors applied to small-object detection.

### Loss functions for small-object detection

Dynamic loss optimization has emerged as a critical strategy for addressing such challenges in small-object detection as low-quality annotations, slow convergence, and insufficient robustness in complex scenarios. Several dynamic loss functions have been proposed, each designed to tackle specific aspects of bounding box regression. GIoU^34^ is introduced to resolve a gradient vanishing problem in cases where the ground truth and predicted boxes have minimal overlap. By using the minimum enclosing box, GIoU helps maintain gradient flow, but it can be susceptible to background interference, particularly in dense environments with many targets. In contrast, DIoU^35^ accelerates convergence by introducing center distance penalties, which improve performance on high-IoU samples. However, this method struggles with targets exhibiting large aspect ratio variations, such as tilted objects seen from unmanned aerial vehicle (UAV) viewpoints. CIoU^36^ further refines the loss by combining penalties for center distance and aspect ratio, achieving a significant $\hbox {mAP}_{50}$ of 43.5 % on the VisDrone2021 dataset. Nevertheless, CIoU is sensitive to low-quality annotations, which can lead to inaccuracies in localization. Many existing methods have inherent limitations. For example, DIoU’s exclusive focus on center distance neglects crucial factors like aspect ratio and orientation. Such methods as DIoU and SIoU rely on static weight allocations, thus failing to adapt to dynamically varying target scales and distributions–issues that are common in UAV-based small-object detection scenarios. Moreover, most conventional IoU-based losses are designed under relatively balanced object-scale distributions and therefore exhibit limited adaptability when dealing with highly dense or extremely small targets. In UAV imagery, small objects often occupy only a few pixels and are easily affected by occlusion, motion blur, and annotation noise. Under such conditions, static geometric penalties may lead to unstable optimization and inaccurate localization refinement. In response to these challenges, recent developments have focused on dynamic and scale-aware losses, which adapt penalty terms based on object scale, shape, and local context^47^. These approaches, including the proposed SD loss, explicitly account for variations in object size and occlusion, thus enhancing localization accuracy for small and partially visible objects.

## Proposed approach

### Lightweight detection framework

**Fig. 1:**
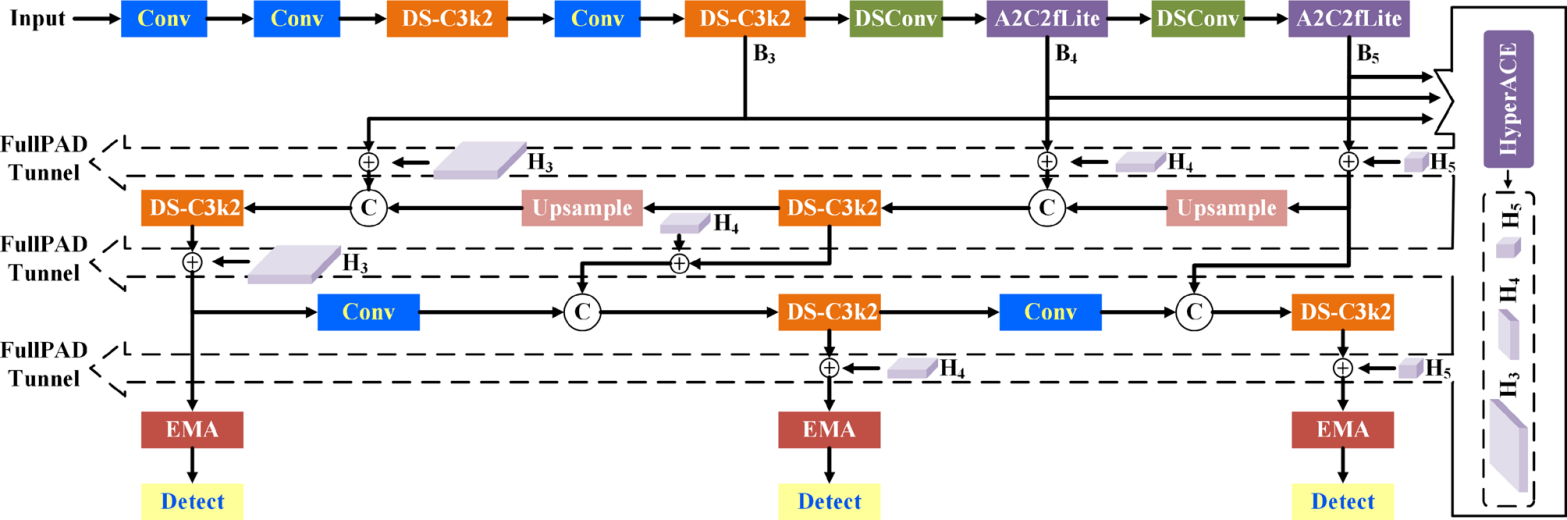
Structure of LSE-YOLO network.

**Fig. 2:**
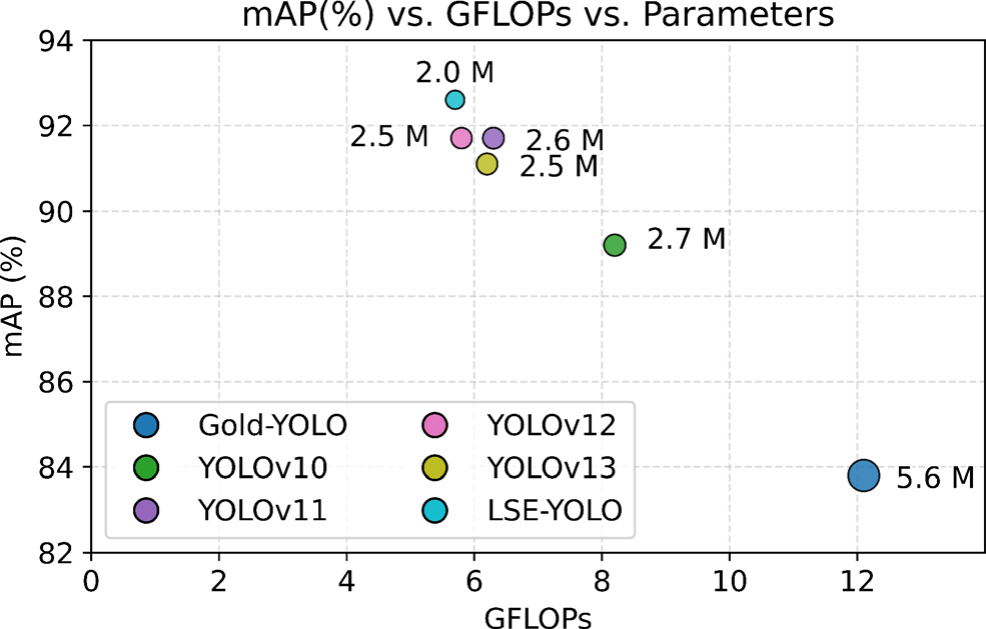
$\hbox {mAP}_{50}$ (%), GFLOPs and parameters of small-object detection methods. In this figure, mAP denotes $\hbox {mAP}_{50}$.

Although YOLOv13 has achieved strong performance in real-time detection tasks, its backbone and neck structures contain redundant parameters and incur high computational cost, which limit its deployment in resource-constrained remote sensing scenarios. To address this issue, its lightweight variant called YOLOv13n has been proposed, significantly reducing model size. However, YOLOv13n faces limitations in detecting remote sensing small-objects due to their insufficient feature representation, background interference, and localization errors. To overcome it, LSE-YOLO is proposed as a lightweight detection approach incorporating SD loss and EMA for remote sensing applications. It integrates three key improvements, as depicted in Fig. 1. First, a lightweight A2C2fLite is newly designed to replace the original A2C2f in the backbone, effectively reducing computational cost and redundant parameters while maintaining strong feature extraction capability. Second, an EMA is innovatively introduced, which is embedded before the last three detection heads in the neck to enhance pixel-level attention allocation and improve the model’s ability to distinguish between targets and background. Finally, the original CIoU is replaced with a novel SD loss function, which improves bounding box regression accuracy by addressing scale sensitivity and localization errors. As shown in Fig. 2, LSE-YOLO achieves $\hbox {mAP}_{50}$ of approximately 92.6 %, while using only 2.0 M parameters and 5.7 GFLOPs. In comparison, YOLOv13 requires 2.5 M parameters and approximately 6.2 GFLOPs, with $\hbox {mAP}_{50}$ around 91.1 %. This demonstrates that our architecture delivers superior accuracy for small-object detection, achieving a better accuracy-efficiency trade-off with lower computational resources and fewer parameters of the closest competitor.

### A2C2fLite

**Fig. 3:**
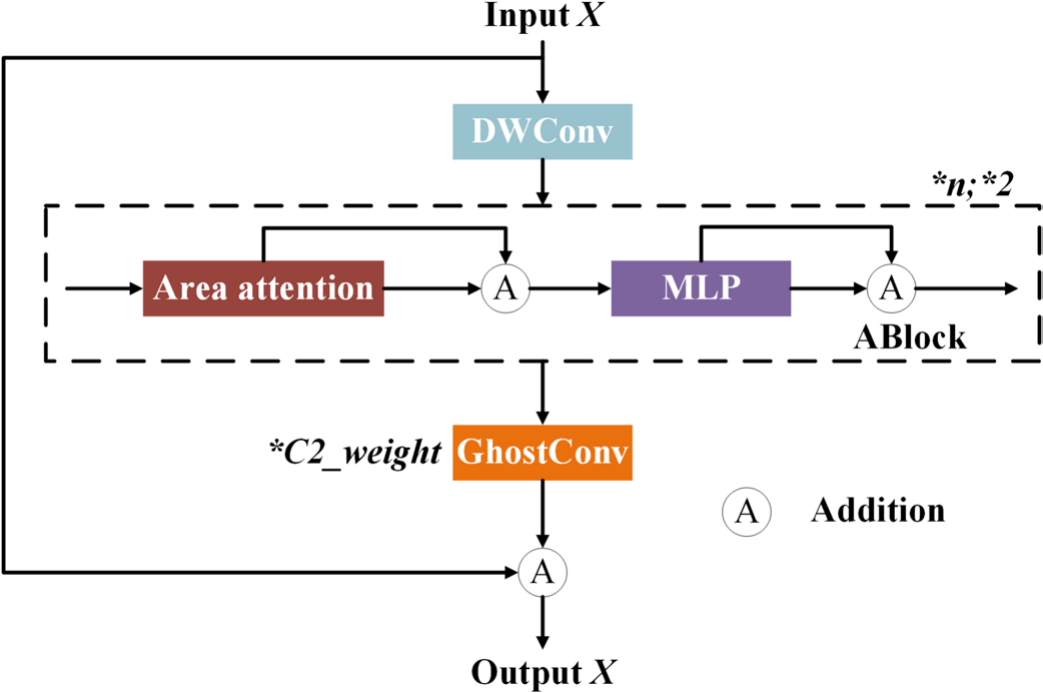
Structure of A2C2fLite.

In order to balance accuracy and efficiency in YOLOv13, we redesign the original A2C2f into a lightweight variant termed A2C2fLite. The A2C2f block relies on standard convolutions and stacked attention modules, which can significantly increase computational overhead, especially in high-resolution feature maps. To reduce it, our A2C2fLite innovatively integrates depthwise separable convolutions, Ghost convolution, simplified attention embedding, and a learnable residual scaling mechanism, thereby achieving a desired trade-off between model complexity and detection performance, as illustrated in Fig. 3.

A2C2fLite is designed as a lightweight alternative to the original A2C2f block in YOLOv13, aiming to reduce computational complexity while maintaining strong feature representation capability. It follows a three-stage process: first, the input feature map is compressed using a depthwise separable convolution, which preserves spatial information but significantly reduces redundant computation. Next, the compressed feature passes through a sequence of lightweight enhancement blocks, where the number of blocks is set to n=1 in the default configuration. Each block adopts a simplified attention embedding based on a single ABlock module, which uses multi-head attention. These blocks enrich the representation with both local and global context information while maintaining low computational cost. The resulting features from multiple stages are then concatenated and transformed by a Ghost convolution, which efficiently reconstructs channel dimensions by combining intrinsic feature maps with additional inexpensive linear operations. To stabilize optimization and preserve input information, a learnable residual scaling mechanism is introduced, implemented as a channel-wise trainable parameter, initialized to 0.1 and jointly optimized during training. It adaptively controls the contribution of the residual branch relative to the transformed features, allowing the network to balance feature refinement and identity preservation. Through this design, A2C2fLite achieves a favorable trade-off between efficiency and accuracy, offering fewer parameters compared to A2C2f, while delivering highly competitive detection performance, as will be shown later.

### Efficient multi-scale attention

**Fig. 4:**
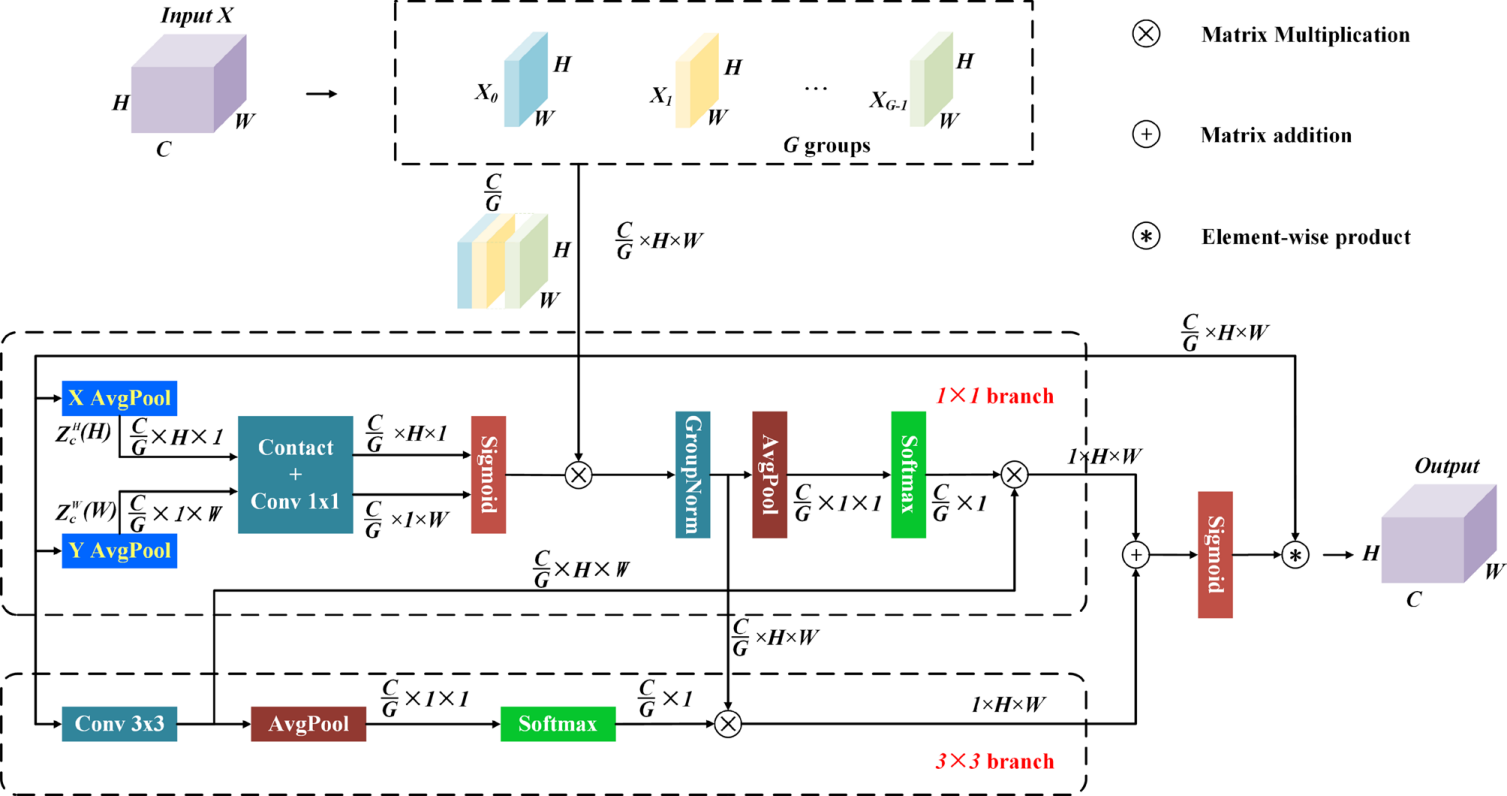
Structure of EMA.

The EMA is integrated into our proposed approach, positioned before the last three detection heads in the neck. It organizes input feature maps by channels and applies parallel convolutional kernels with varying receptive fields to capture multi-scale contextual information for remote sensing small objects. A two-dimensional global average pooling operation is used to encode global cues and pixel-level dependencies, enhancing spatial and channel correlations. This mechanism improves object feature discrimination, particularly in noisy environments, strengthening the detection of small remote sensing objects. The EMA network structure is shown in Fig. 4.

Following the design of EMA in^11^, the input feature map $\textbf{X} \in \mathbb {R}^{C \times H \times W}$ extracted from the backbone is first partitioned into *G* groups along the channel dimension, resulting in a set of sub-feature maps:

1 $$\begin{aligned} \textbf{X} = [\textbf{X}_0, \textbf{X}_1, \dots , \textbf{X}*i, \dots , \textbf{X}*{G-1}] \end{aligned}$$

where each sub-feature $\textbf{X}_i \in \mathbb {R}^{\frac{C}{G} \times H \times W}$, and *H*, *W*, and *C* denote the height, width, and number of channels, respectively.

These grouped features are then processed by two parallel branches. In the first branch, a *1 × 1* convolution is applied, followed by two parallel operations: global average pooling along the horizontal and vertical directions, respectively. The resulting features are further normalized using Group Normalization, and subsequently refined through average pooling and softmax operations. This branch effectively captures long-range spatial dependencies while integrating channel-wise information, producing a compact representation of size $G \times 1 \times 1$.

2 $$\begin{aligned} Z_{c}=\frac{1}{HW}\sum _{i=1}^{H}\sum _{j=1}^{W}x_{c}(i,j), \end{aligned}$$

where $\textsc {Z}_{c}$ represents the feature of the *c*-th channel after two-dimensional global average pooling, and $x_{c}(i,j)$ represents the input feature at position (*i*, *j*) in the spatial dimension of the *c*-th channel

In the second branch, following the Softmax operation, the obtained feature map is multiplied with the sub-feature maps processed by a *3 × 3* convolution, producing an initial spatial attention map of size $1 \times H \times W$. Similar to the *1 × 1* branch, the *3 × 3* branch also captures spatial dependencies through parallel processing. Furthermore, the output of the *3 × 3* branch is combined via matrix multiplication with the feature map from the *1 × 1* branch after group normalization, resulting in another spatial attention map of the same dimension. This process enhances the interaction of spatial information across different regions. Finally, the two spatial attention maps are aggregated and passed through a Sigmoid activation function to generate the final attention weights. These weights are then applied to the original feature map through element-wise multiplication, enabling the network to focus on more informative regions while suppressing irrelevant background features.

### Scale-based dynamic loss

To improve the stability of bounding box regression for small-object detection, the SD loss adopted in LSE-YOLO is built upon a modified CIoU formulation with a scale-dependent modulation term. Unlike conventional IoU-based loss functions such as CIoU and DIoU, the proposed method introduces a dynamic coefficient $\beta _B$ to adjust regression sensitivity according to object scale. Specifically, $\beta _B$ is computed based on the ground-truth bounding box area normalized by a predefined maximum reference area and further scaled by the feature map resolution:

3 $$\begin{aligned} \beta _B = \frac{w_{gt} \cdot h_{gt} \cdot \delta }{81} \end{aligned}$$

To ensure training stability, $\beta _B$ is constrained within a predefined range using a clipping operation with threshold *δ = 0.5*. Based on this coefficient, the final SD loss is implemented by directly modulating the CIoU-based regression term, where the scale-aware factor is embedded into both the IoU consistency and geometric penalty components. This allows the loss function to adaptively adjust the contribution of localization and shape constraints according to object size. Compared with conventional IoU-based losses, the proposed formulation introduces only lightweight linear operations and avoids additional computational overhead, making it suitable for real-time remote-sensing object detection tasks.

## Experiments and discussion

### Datasets and metrics

The experiments are conducted on the NWPU VHR-10 dataset^48^, which is widely adopted for small-object detection in aerial and remote sensing applications. The dataset contains 650 images with objects and 150 background images, resulting in a total of 800 images. The annotated objects are characterized by small size, varying orientations, complex backgrounds, and high intra-class similarity, making the dataset a representative benchmark for evaluating detection performance in challenging scenarios. For experimental evaluation, the dataset is divided into training, validation, and testing sets with a ratio of 7:2:1, ensuring balanced coverage of all object categories across different subsets.

In this work, the performance of LSE-YOLO is evaluated by using precision (P), recall (R), $\hbox {mAP}_{50}$, and $\hbox {mAP}_{50-95}$, as well as model complexity indicators including GFLOPs and number of parameters. The IoU measures the overlap between predicted bounding boxes and ground truth annotations, and different IoU thresholds are used to determine prediction correctness. Specifically, a prediction is considered correct if its IoU with the ground truth exceeds the threshold; otherwise, it is deemed incorrect. $\hbox {mAP}_{50}$ summarizes detection performance under IoU = 0.5, while $\hbox {mAP}_{50-95}$ provides a stricter evaluation by averaging results across multiple IoU thresholds, thus better reflecting localization quality. P and R further evaluate the model’s ability to correctly identify true positives while minimizing false positives and false negatives. GFLOPs quantify computational cost, while the number of parameters reflects model size and memory footprint. Collectively, these metrics provide a comprehensive assessment of both detection performance and model efficiency.

4 $$\begin{aligned} P&= \frac{TP}{TP+FP} \end{aligned}$$

5 $$\begin{aligned} R&= \frac{TP}{TP+FN} \end{aligned}$$

6 $$\begin{aligned} \text {AP}&= \int _{0}^{1} P(R)\,dR \end{aligned}$$

7 $$\begin{aligned} \text {mAP}&= \frac{1}{k}\sum _{i=1}^{k}\text {AP}_{i} \end{aligned}$$

where *TP* stands for true positive samples, *FP* for false positive examples, and *FN* for false negative samples ^49^. *k* is the total number of labels, and AP is the average precision of a single class label. In this work, $\hbox {mAP}_{50}$ denotes the mean average Precision computed at IoU threshold 0.5, while $\hbox {mAP}_{50-95}$ denotes the average mAP over multiple IoU thresholds from 0.5 to 0.95.

### Implementation details

All experiments are implemented using the PyTorch deep learning framework and conducted on a server equipped with an Intel Xeon Gold 6430 CPU and an NVIDIA RTX 4090 GPU. CUDA 12.4 is used for acceleration. For fair comparison, all models are trained under identical settings using the same codebase and dataset split. The input resolution is set to *640 × 640*, and the total training epochs are 300 with a batch size of 16. The optimizer is automatically selected by the training framework, with an initial learning rate of 0.01, momentum of 0.937, and weight decay of 0.0005. A cosine learning rate schedule with a warmup strategy is adopted to stabilize early-stage training. To improve reproducibility, we explicitly fix the random seed to 0 and enable deterministic training. Data augmentation strategies, including mosaic, mixup, HSV augmentation, and random flipping, follow the default configuration of the YOLOv13 framework to ensure consistent training protocols across all compared methods. For inference evaluation, a confidence threshold of 0.25 and IoU threshold of 0.7 are used, and all results are reported under identical NMS settings to ensure fairness.

### Ablation experiment

The proposed LSE-YOLO model introduces three improvements: A2C2fLite, SD loss, and EMA. They are integrated into the baseline YOLOv13n framework to enhance its detection performance. Ablation experiments are conducted and the results are summarized in Table 1. A2C2fLite effectively reduces redundant parameters and computational cost while improving feature extraction efficiency. Compared with the baseline, it reduces 6.2 to 5.7 GFLOPs and 2.5 M to 2.0 M parameters, improving accuracy from 90.2 % to 92.8 % but reducing R and $\hbox {mAP}_{50}$ slightly. This decline in recall can be attributed to the reduced feature redundancy introduced by the lightweight design. By replacing standard convolutions with depthwise separable and Ghost convolutions, A2C2fLite improves efficiency but weakens sensitivity to low-contrast or partially occluded small objects, which leads to an increase in false negatives. The use of SD loss improves the bounding box regression accuracy by optimizing the overlap calculation between predicted and ground truth boxes. When combined with A2C2fLite, it enhances the precision of localization, leading to an improvement of 3.1 % in P and 0.6 % in $\hbox {mAP}_{50}$ over the baseline but lowering R by 1.2 %. In addition to $\hbox {mAP}_{50}$, $\hbox {mAP}_{50-95}$ exhibits a different trend compared to the baseline. Although SD loss improves regression quality, the introduction of A2C2fLite leads to a slight degradation in $\hbox {mAP}_{50-95}$ relative to the baseline. This is mainly because lightweight feature compression reduces fine-grained spatial details, which are more critical under high IoU thresholds. As a result, the model prioritizes detection efficiency and coarse localization accuracy, while extremely precise bounding box refinement becomes more challenging. EMA provides stable parameter updates during training, enhancing model generalization and robustness. EMA helps partially mitigate this issue by stabilizing feature representations across training iterations, which improves consistency of object localization and reduces instability in hard samples. However, its effect is more pronounced on recall recovery rather than fine-grained localization refinement, which explains why $\hbox {mAP}_{50-95}$ does not surpass the baseline significantly. When integrated with A2C2fLite and SD loss, our full model achieves the best results, with P, R, and $\hbox {mAP}_{50}$ reaching 93.6 %, 86.6 %, and 92.6 %, respectively. Although $\hbox {mAP}_{50-95}$ does not show a significant improvement over the baseline, it remains competitive (59.7%), indicating that the proposed method maintains acceptable high-IoU performance while significantly improving overall detection accuracy at $\hbox {mAP}_{50}$. This trade-off is reasonable in UAV-based small-object detection, where detection robustness and recall are often prioritized over extremely tight localization. Overall, $\hbox {mAP}_{50}$ reflects detection robustness under moderate IoU constraints, while $\hbox {mAP}_{50-95}$ reflects fine-grained localization accuracy. The observed trade-off demonstrates that the proposed modules improve detection reliability and efficiency, at the cost of slightly reduced high-precision localization sensitivity, which is a common and acceptable compromise in lightweight UAV detection models.

**Table 1:** Ablation experiment results. Best results in **bold**, runner-ups underlined.

Method	P (%)	R (%)	$\hbox {mAP}_{50}$ (%)	$\hbox {mAP}_{50-95}$ (%)	GFLOPs	Parameters (M)
Baseline	90.2	**86.6**	91.1	**62.0**	6.2	2.5
+ A2C2fLite	92.8	84.2	90.1	58.5	**5.7**	**2.0**
+ SD loss + A2C2fLite	93.3	85.4	91.7	58.2	**5.7**	**2.0**
+ SD loss + A2C2fLite + EMA	**93.6**	**86.6**	**92.6**	59.7	**5.7**	**2.0**

### IoU-based loss vs. SD loss

To assess the effectiveness of the introduced SD loss in the LSE-YOLO model, comparative experiments are conducted with commonly used IoU-based loss functions, including CIoU^36^, DIoU^35^, GIoU^34^, and Focaler-IoU (FIoU)^50^, as shown in Table 2. The results indicate that CIoU achieves relatively high P (94.5 %) but limited R and $\hbox {mAP}_{50}$ performance. DIoU and GIoU improve recall to some extent, but both exhibit lower precision, with DIoU dropping P significantly to 85.6 %. FIoU shows the weakest overall performance, with the lowest $\hbox {mAP}_{50}$ of 88.5 %. In addition to $\hbox {mAP}_{50}$, $\hbox {mAP}_{50-95}$ provides a stricter evaluation of localization quality under higher IoU thresholds. From Table 2, CIoU achieves relatively strong performance on $\hbox {mAP}_{50-95}$ (59.0%), indicating stable localization under moderate overlap conditions. However, DIoU and GIoU decrease to 57.1% and 57.5%, respectively, reflecting reduced robustness under stricter IoU constraints. FIoU further degrades to 58.4%, demonstrating limited generalization to high-precision localization. In contrast, the proposed SD loss achieves the best balance across all evaluation metrics, reaching 93.6 %, 86.6 %, and 92.6 % in terms of P, R, and $\hbox {mAP}_{50}$, respectively. It also obtains the highest $\hbox {mAP}_{50-95}$ of 59.7%, demonstrating consistent improvements not only in coarse detection accuracy but also in fine-grained bounding box localization. Compared with other IoU-based losses, it not only preserves high precision but also significantly improves recall and overall detection accuracy. These results indicate that SD loss enhances both detection confidence ($\hbox {mAP}_{50}$) and localization precision ($\hbox {mAP}_{50-95}$), leading to a more robust regression behavior in small-object detection scenarios.

**Table 2:** Comparison experiment results of different IoU-based loss functions. Best results in **bold**, runner-ups underlined.

Method	P (%)	R (%)	$\hbox {mAP}_{50}$ (%)	$\hbox {mAP}_{50-95}$ (%)
LSE-YOLO + CIoU (baseline)	**94.5**	84.8	91.3	59.0
LSE-YOLO + DIoU	85.6	**86.8**	90.1	57.1
LSE-YOLO + GIoU	90.4	84.6	90.0	57.5
LSE-YOLO + FIoU	90.8	83.9	88.5	58.4
LSE-YOLO + SD loss	93.6	86.6	**92.6**	**59.7**

**Fig. 5:**
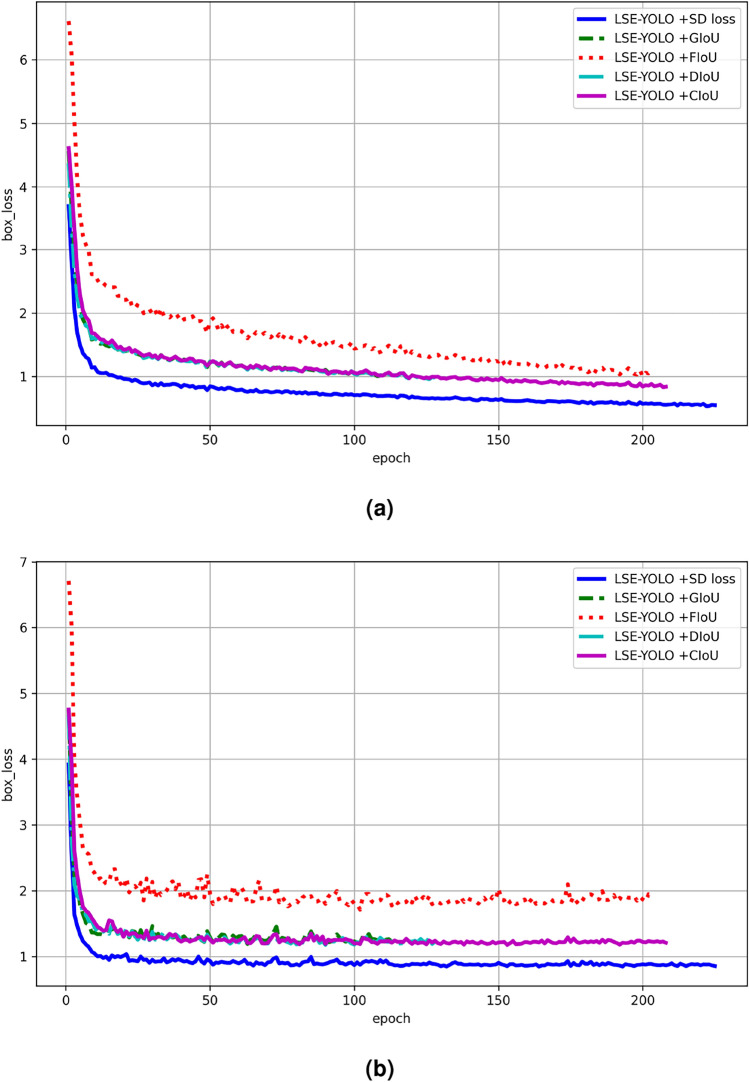
Loss comparison of different IoU-based loss functions. (**a**) Training loss. (**b**) Validation loss.

Fig. 5a, b show the training and validation loss curves of LSE-YOLO when five IoU variants are used as localization losses. During the whole training process, SD loss (blue curve) converges the fastest and stabilizes at the lowest loss value on both figures, indicating that it provides the strongest and most stable supervision for bounding-box regression. Among IoU-based losses, GIoU, DIoU, and CIoU exhibit nearly identical loss curves, with their training and validation losses following similar trends. This suggests that these methods offer comparable performance, showing no significant differences in optimization speed or final loss values. FIoU demonstrates a similar descent speed but eventually plateaus at higher values. These results demonstrate that SD loss not only accelerates optimization but also generalizes better.

### Hyperparametric experiment

To investigate the effect of channel grouping in the EMA module, we conduct a series of hyperparameter studies on the NWPUVHR-10 dataset. Different group numbers are evaluated, and the corresponding results are reported in Table 3. The experimental results indicate that the model achieves the best performance when the number of groups is set to *G = 8*, yielding P = 93.6%, R = 86.6%, and $\hbox {mAP}_{50}$ = 92.6%. Increasing the number of groups allows the attention mechanism to capture more fine-grained channel-wise information, which contributes to performance improvement. However, when *G* becomes excessively large, the interaction between groups is weakened, leading to a degradation in detection accuracy. In addition to $\hbox {mAP}_{50}$, $\hbox {mAP}_{50-95}$ further reflects the influence of group settings on localization quality under stricter IoU thresholds. As shown in Table 3, the model achieves the best $\hbox {mAP}_{50-95}$ (59.7%) when *G = 8*, while both smaller and larger group settings result in lower values, indicating suboptimal balance between feature diversity and cross-group interaction. Based on these observations, we set *G = 8* in the EMA module for subsequent experiments, as it provides the best trade-off between detection accuracy and localization robustness.

**Table 3:** EMA module hyperparametric experiments. Best results in **bold**, runner-ups underlined.

Group number setting	P (%)	R (%)	$\hbox {mAP}_{50}$ (%)	$\hbox {mAP}_{50-95}$ (%)
Group = 2	89.8	86.3	90.3	58.4
Group = 4	91.3	85.5	90.3	58.5
Group = 8	**93.6**	**86.6**	**92.6**	**59.7**
Group = 16	91.5	85.5	90.0	58.0
Group = 32	91.4	85.2	90.7	58.8

### Comparative experiments on attentional mechanisms

To evaluate the effectiveness of EMA in the proposed model, various attention mechanisms are introduced into LSE-YOLO for comparative experimentation, as detailed in Table 4. The experimental results indicate that although CBAM^32^ and SE^31^ improve recall to some extent, their overall performance in terms of P and $\hbox {mAP}_{50}$ is suboptimal, with CBAM achieving only 86.1 % in P and 89.7 % in $\hbox {mAP}_{50}$, while SE reaching 89.8 % in P and 88.4 % in $\hbox {mAP}_{50}$. The CA^33^ achieves relatively balanced results, with P = 92.7 %, R = 84.9 %, and $\hbox {mAP}_{50}$ = 90.8 %, but lags behind EMA. By contrast, EMA significantly enhances detection performance, yielding P = 93.6 %, R = 86.6 %, and $\hbox {mAP}_{50}$ = 92.6 %. In addition to $\hbox {mAP}_{50}$, EMA also improves $\hbox {mAP}_{50-95}$ to 59.7%, which is consistently higher than most competing attention mechanisms. This indicates that EMA not only improves coarse-level detection accuracy (IoU=0.5) but also enhances localization quality under stricter IoU thresholds, demonstrating stronger bounding-box refinement capability. Compared with the baseline model without attention, EMA improves $\hbox {mAP}_{50}$ from 91.7% to 92.6% and maintains competitive performance in $\hbox {mAP}_{50-95}$, showing that the gains are consistent across both loose and strict evaluation metrics. These results not only surpass the LSE-YOLO without attention but also outperform other attention mechanisms across all evaluation metrics. This confirms that EMA provides superior feature refinement and more effective attention allocation, thereby improving the overall detection capability of LSE-YOLO. In terms of efficiency, EMA is designed as a lightweight module with negligible computational overhead compared to convolutional backbone operations, and thus introduces only a minor increase in inference latency. In practical real-time deployment scenarios, such as UAV-based detection, this trade-off is considered acceptable because the performance gain in both $\hbox {mAP}_{50}$ and $\hbox {mAP}_{50-95}$ is achieved without significantly affecting the overall inference speed. Therefore, the improvement is justified in resource-constrained applications where both accuracy and efficiency are required.

**Table 4:** Comparison experiment results of different attention mechanisms. Best results in **bold**, runner-ups underlined.

Model	Attention Module	P (%)	R (%)	$\hbox {mAP}_{50}$ (%)	$\hbox {mAP}_{50-95}$ (%)
LSE-YOLO	–	93.3	85.4	91.7	58.2
LSE-YOLO	CBAM	86.1	**87.1**	89.7	57.6
LSE-YOLO	SE	89.8	85.6	88.4	58.7
LSE-YOLO	CA	92.7	84.9	90.8	**59.7**
LSE-YOLO	EMA	**93.6**	86.6	**92.6**	**59.7**

**Fig. 6:**
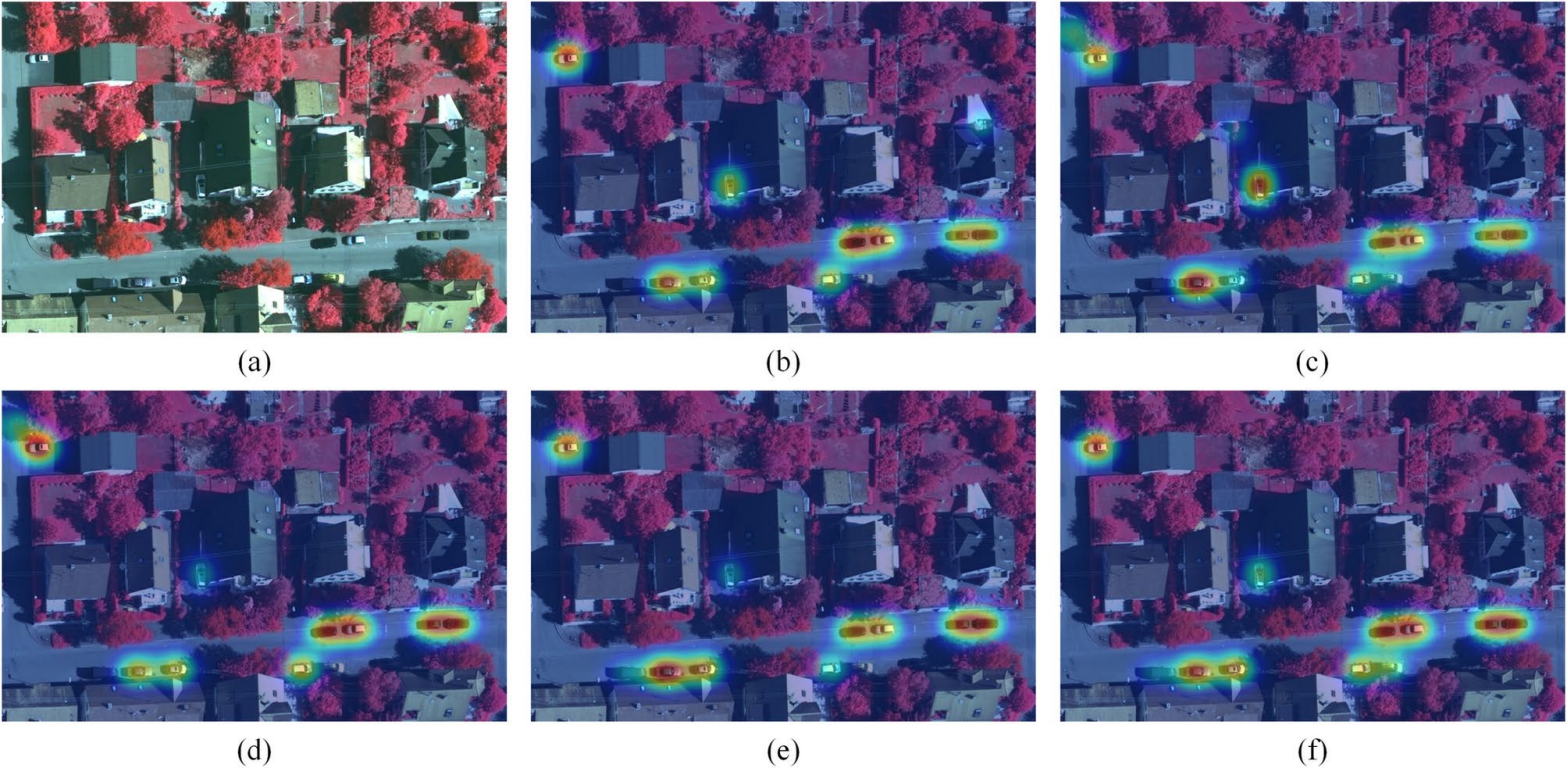
Heatmaps of different attention mechanisms. (**a**) Original picture, (**b**) Without attention (LSE-YOLO), (**c**) LSE-YOLO + SE, (**d**) LSE-YOLO + CA, (**e**) LSE-YOLO + CBAM, and (**f**) LSE-YOLO + EMA.

Fig. 6 shows the heatmaps of different attention mechanisms when facing an remote sensing scene with extremely small targets. The LSE-YOLO without any attention in Fig. 6b exhibits diffuse responses, where the target intensity is almost drowned in cluttered background. SE in Fig. 6c slightly amplifies the object region. Yet its global channel recalibration neglects precise spatial cues, leaving considerable noise around the edges. CA in Fig. 6d introduces accurate position encoding. However, the single-scale kernel limits its receptive field, so only part of the target is highlighted and adjacent textures are still activated. CBAM in Fig. 6e sequentially refines both channel and spatial weights, but the two independent branches lack cross-dimension interaction, resulting in fragmented focus and incomplete suppression of distractors. By contrast, EMA in Fig. 6f establishes long-range dependencies in a group-wise manner and fuses multi-scale context within a single lightweight module. Consequently, the heat-map presents a compact, high-intensity blob exactly covering the target while background responses are almost erased, verifying that EMA is more capable of capturing diminutive yet critical objects under complex remote sensing backgrounds than its peers.

### Comparison of different object detection models

To evaluate the effectiveness of the proposed LSE-YOLO, we conduct extensive experiments and compare it with several competitive object detection methods, including both CNN-based and Transformer-based ones. The CNN-based baselines include Gold-YOLO^51^, YOLOv5, YOLOv8, YOLOv10, YOLOv11, YOLOv12, YOLOv13, and RT-Detr^52^. The last one represents a typical Transformer-based detector. All models are trained and tested with the same dataset settings and training protocols to ensure fair comparison. As shown in Table 5, LSE-YOLO achieves P = 93.6 %, R = 86.6 %, and $\hbox {mAP}_{50}$ = 92.6 %, outperforming previous YOLO variants such as YOLOv5, YOLOv8, YOLOv10, YOLOv11, YOLOv12, and YOLOv13 in terms of detection accuracy. In particular, compared with widely used baselines such as YOLOv5 and YOLOv8, LSE-YOLO achieves consistent improvements on both $\hbox {mAP}_{50}$ and $\hbox {mAP}_{50-95}$, demonstrating its stronger capability in both object classification confidence and fine-grained localization accuracy. Compared with RT-Detr, which achieves $\hbox {mAP}_{50}$ = 91.4 % with significantly higher computational cost (103.5 GFLOPs) and parameter count (32.0 M), LSE-YOLO demonstrates a favorable trade-off between accuracy and efficiency, consuming only 5.7 GFLOPs and 2.0 M parameters. In addition, $\hbox {mAP}_{50-95}$ further reflects the robustness of LSE-YOLO under stricter IoU thresholds. Although transformer-based RT-DETR achieves the highest $\hbox {mAP}_{50-95}$ (62.4%), it does so at a significantly higher computational cost. In contrast, LSE-YOLO maintains competitive $\hbox {mAP}_{50-95}$ (59.7%) while requiring substantially fewer GFLOPs and parameters, indicating a more favorable efficiency–accuracy trade-off. These results suggest that LSE-YOLO not only maintains strong detection capability in remote sensing scenarios but also achieves superior computational efficiency, making it particularly suitable for resource-constrained deployment environments. Overall, it outperforms both conventional CNN-based detectors and computationally expensive Transformer-based methods, demonstrating the practical advantages of combining lightweight design with adaptive attention and loss mechanisms.

**Table 5:** Comparison experiments result of different object detection models. Best results in **bold**, runner-ups underlined.

Method	P (%)	R (%)	$\hbox {mAP}_{50}$ (%)	$\hbox {mAP}_{50-95}$ (%)	GFLOPs	Parameters
Gold-YOLO	90.6	79.0	83.8	49.4	12.1	5.6 M
YOLOv10	88.3	84.1	89.2	59.6	8.2	2.7 M
RT-DETR	89.7	**89.0**	91.4	62.4	103.5	32.0 M
YOLOv5	89.4	86.5	90.7	59.4	7.1	2.5 M
YOLOv8	90.7	87.6	91.9	60.1	8.1	3.0 M
YOLOv11	91.0	87.1	91.7	**62.5**	6.3	2.6 M
YOLOv12	91.8	86.7	91.7	61.6	5.8	2.5 M
YOLOv13	90.2	86.6	91.1	62.0	6.2	2.5 M
LSE-YOLO	**93.6**	86.6	**92.6**	59.7	**5.7**	**2.0 M**

### Per-class evaluation on NWPU VHR-10 dataset

As shown in Table 6, the proposed LSE-YOLO achieves consistently high detection performance across most categories in the NWPU VHR-10 dataset. In particular, high-contrast and well-structured objects such as airplanes, baseball diamonds, and tennis courts obtain strong $\hbox {mAP}_{50}$ scores exceeding 98%, demonstrating the model’s effectiveness in capturing regular geometric structures. In contrast, relatively complex and densely distributed categories such as storage tanks and bridges show lower performance, especially in $\hbox {mAP}_{50-95}$. This is mainly due to severe scale variations, occlusions, and background clutter, which make precise localization more challenging under stricter IoU thresholds. Ship and basketball court categories also exhibit moderate performance degradation compared with simpler targets, indicating that the model is still sensitive to intra-class variation and small object ambiguity in cluttered maritime and urban scenes. Overall, the results demonstrate that LSE-YOLO performs robustly across most aerial object categories, while performance variations across classes are primarily influenced by object density, shape variability, and background complexity. This further validates the effectiveness of the proposed lightweight design in remote sensing object detection scenarios.

**Table 6:** Class-wise detection results of LSE-YOLO on the NWPU VHR-10 dataset.

Categories	P (%)	R (%)	$\hbox {mAP}_{50}$ (%)	$\hbox {mAP}_{50-95}$ (%)
Airplane	97.9	99.2	99.4	71.5
Ship	89.4	75.6	86.4	61.2
Storage tank	81.3	83.7	81.9	25.1
Baseball diamond	94.4	96.7	98.4	76.1
Tennis court	96.0	96.1	98.8	72.3
Basketball court	90.0	76.3	87.1	60.0
Ground track field	95.3	100.0	97.5	82.2
Harbor	97.9	88.6	94.9	57.8
Bridge	97.7	66.7	87.9	31.1
Vehicle	95.9	82.7	93.5	59.7

### Computational complexity and inference efficiency

To further evaluate the practical deployment capability of the proposed method, additional runtime benchmarks are conducted under the same hardware and software environment. All experiments are performed on an NVIDIA RTX 4090 GPU with an input resolution of *640 × 640* and batch size 1. To ensure stable and fair evaluation, each benchmark is repeated five times after 100 warm-up iterations, and the average results are reported. In addition to GFLOPs and parameter count, which mainly reflect theoretical computational complexity, latency, Frames Per Second (FPS), and memory consumption are further introduced to evaluate practical inference efficiency. Specifically, latency measures the average inference time per image, FPS indicates real-time processing capability, and memory consumption reflects runtime resource requirements during inference.

As shown in Table 7, the proposed method achieves competitive real-time inference performance while maintaining lightweight model characteristics. Compared with the baseline model, the proposed approach reduces the number of parameters from 2.5M to 2.0M and decreases computational complexity from 6.2 GFLOPs to 5.7 GFLOPs. In terms of deployment efficiency, the proposed method achieves a lower inference latency of 14.62 ms, outperforming the baseline latency of 18.14 ms. Meanwhile, the inference speed increases significantly from 55.52 FPS to 68.43 FPS, demonstrating improved real-time processing capability. Although additional feature enhancement modules are introduced, the GPU memory consumption remains nearly unchanged, with only a marginal increase of approximately 1 MB.

These results indicate that the proposed method maintains favorable computational efficiency and runtime performance while improving detection accuracy, demonstrating its potential applicability for resource-constrained remote-sensing and UAV-based real-time detection tasks. Although edge-device deployment experiments are not included in the current study, the reduced computational complexity and stable runtime behavior suggest promising deployment potential for embedded platforms in future work.

**Table 7:** Computational complexity and inference efficiency.

Model	Parameters *↓*	GFLOPs *↓*	Latency (ms) *↓*	FPS *↑*	Memory (MB) *↓*
Baseline	2.5 M	6.2	18.1	55.5	203.1
Ours	2.0 M	5.7	14.6	68.4	204.1

## Visualization of detection results

**Fig. 7:**
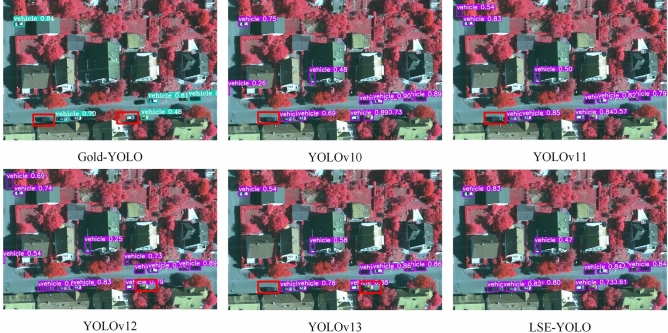
Visualization of vehicle detection results among Gold-YOLO, YOLOv10, YOLOV11, YOLOv12, YOLOV13 and LSE-YOLO.

**Fig. 8:**
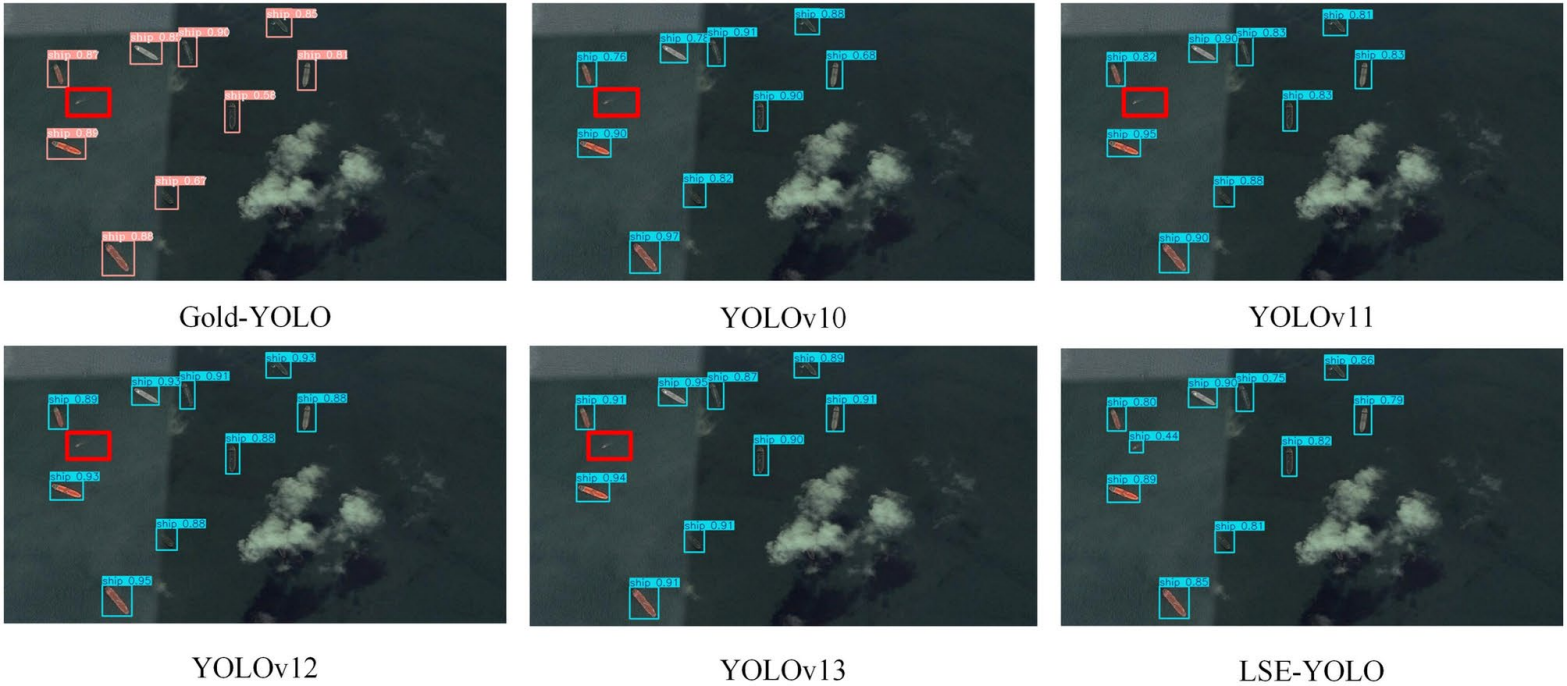
Visualization of ship detection results among Gold-YOLO, YOLOv10, YOLOV11, YOLOv12, YOLOV13 and LSE-YOLO.

To visually compare the detection performance of LSE-YOLO with its peers, several representative images from the NWPUVHR-10 dataset are presented in Figs 7 and 8. They illustrate that Gold-YOLO, YOLOv10, YOLOv11, YOLOv12, and YOLOv13, occasionally suffer from missed detection or low-confidence predictions, particularly for small or densely packed vehicles and ships, as indicated by the red rectangles. In contrast, LSE-YOLO demonstrates superior detection performance, successfully identifying most vehicles and ships with higher confidence scores. The improvements can be attributed to EMA that enhances pixel-level attention allocation, and SD loss that improves bounding box regression for small objects. Overall, the visualization confirms that LSE-YOLO not only reduces missed detections but also improves localization accuracy, highlighting its effectiveness and robustness in remote sensing small-object detection scenarios.

## Conclusion

This work presents LSE-YOLO, a lightweight and efficient object detection framework for remote-sensing small-object detection. The proposed method is built upon a redesigned lightweight backbone, A2C2fLite, and further integrates existing EMA attention and SD loss to improve feature representation and localization performance. Experimental results on the NWPU VHR-10 dataset demonstrate that LSE-YOLO achieves competitive detection accuracy while significantly reducing computational cost and model complexity compared with representative YOLO variants. The results indicate that the proposed design is particularly effective for small and densely distributed object detection, while maintaining a favorable balance between accuracy and efficiency. It should be noted that the current evaluation is mainly conducted on a single benchmark dataset. Therefore, the generalization capability of the proposed method across different remote-sensing scenarios remains to be further validated. Future work will focus on extending the evaluation to larger and more diverse remote-sensing datasets, improving cross-scene robustness, and further optimizing the efficiency of the backbone and feature enhancement modules. These directions are expected to enhance the applicability of lightweight detection models in remote-sensing and UAV-based vision tasks.

## Data Availability

All relevant data are within the manuscript. Additionally, the dataset underlying the results of this study are available from public repositories. Specifically, the NWPU VHR-10 dataset is available at https://github.com/Gaoshuaikun/NWPU-VHR-10.
